# Test–retest reliability of kinematic and kinetic parameters during dual-task stair walking in the elderly

**DOI:** 10.3389/fphys.2023.1177159

**Published:** 2023-05-09

**Authors:** Yue Li, Ning Yu, Cui Zhang, Qipeng Song, Jiangna Wang, Wei Sun

**Affiliations:** ^1^ College of Sports and Health, Shandong Sport University, Jinan, China; ^2^ School of Science, Shandong Jianzhu University, Jinan, China; ^3^ Shandong Institute of Sports Science, Jinan, China

**Keywords:** dual-task, stair walking, reliability, kinematics, kinetics

## Abstract

**Objective:** This study aims to evaluate the test–retest reliability of kinematics and kinetics during single and dual-task stair walking in the elderly.

**Methods:** Fifteen healthy elderly adults were recruited. Kinematic and kinetic parameters were measured using an infrared motion analysis system (Vicon, Oxford Metrics Ltd., Oxford, United Kingdom) and force platforms (Switzerland, Kistler 9287BA and 9281CA). Participants were tested under single-task and dual-task (serial 3 subtractions or carrying a cup of water) conditions. Each participant completed two sessions on two separate days with a 1-week interval. Intraclass correlation coefficients (ICC), Pearson correlation coefficient (r), and Bland–Altman plot were used to assess the reliability of stair walking.

**Results:** When ascending stairs, the ICC of kinematics and kinetics ranged from fair to excellent (ICC = 0.500–0.979) in the single and dual tasks, except for step length (ICC = 0.394) in the single task. The r value of kinematics and kinetics ranged from 0.704 to 0.999. When descending stairs, the ICC of kinematics and kinetics ranged from good to excellent (ICC = 0.661–0.963), except for min hip moment (ICC = 0.133) and min ankle moment (ICC = 0.057) in the manual task. The r value of kinematics and kinetics ranged from 0.773 to 0.960 in the single and dual tasks. In the Bland–Altman plots, all the zero values and most of the dots fell in the 95% confidence interval, and the mean difference was found to be close to zero for all the parameters during stair walking.

**Conclusion:** These results obtained from this study show the good test-retest reliability of step cadence, step speed, and step width during single- and dual-task stair walking in the elderly, and the poor reliability of step length during ascending stairs. All the kinetic parameters, including min hip moment, max knee moment, and min ankle moment, had good test-retest reliability during single- and dual-task stair walking, but min hip moment and min ankle moment had poor reliability during manual-task descending stair. These results may help researchers in the assessment of biomechanics of dual-task stair walking in the elderly and to interpret the effect of interventions in this population.

## 1 Introduction

Approximately 30% of elderly people aged 65 years or older fall each year (Chippendale and Raveis, 2017). Falling during stair walking is a common cause of musculoskeletal injuries, hip fractures (Kim et al., 2022), craniocerebral injury, and even mortality (Clark and Arnold, 2021; Kim and Kim, 2022), leading to a substantial economic burden for family and society (Moreland et al., 2020). Stair walking is a difficult and challenging task for elderly people due to the deterioration of their physical function (Ito et al., 2020). More than half of all reported fall injuries occur when walking on stairs or slopes (Sheehan and Gottschall, 2012) (Blazewick et al., 2018).

The test–retest reliability of kinematic and kinetic parameters during single-task stair walking or level walking has been demonstrated in literature (Mentiplay and Clark, 2018); (Jarvis et al., 2021); (Leitner et al., 2011). As a previous study demonstrated, the test-retest reliability of all gait models was mostly good to excellent during level walking (Mentiplay and Clark, 2018). And the test-retest reliability of walking speed is excellent (ICC = 0.93–0.94) when using the Gait Box during level walking (Jarvis et al., 2021). Under a single task, stair walking had moderate to good reliability, with intraclass correlation coefficients (ICC) ranging from 0.537 to 0.836 and coefficients of variation (CV) ranging from 2.51% to 6.51% in vertical ground reaction forces (Leitner et al., 2011). In the single-task walking condition, all the components of the measure suggest good to excellent reliability (Brusola et al., 2021).

Dual-task interference could further impair gait performance and increase the risk of fall during stair walking in older people (Vallabhajosula et al., 2015). The dual-task (DT) paradigm leads to the competition of attention resources available and will influence the posture control of the elderly in stair negotiation (Simoni et al., 2013; Bianca et al., 2021). This paradigm refers to the performance of a cognitive task (CT) and manual task (MT) simultaneously, such as talking, calculating, and texting while moving an object to another place or carrying a tray with cups of water during stair walking (Paul et al., 2009; Asai et al., 2014; Belur et al., 2020). Dual tasks could significantly decrease gait performance and lower limb joint movement (Wang et al., 2022), reduce the gait speed, and increase the step width and body sway during stair walking (Madehkhaksar and Egges, 2016; Zhang et al., 2018).

However, the reliability of stair walking under a dual-task condition in the elderly is currently unknown. This study aimed to assess the test–retest reliability of kinematic and kinetic parameters in stair negotiation under single and dual-task conditions. Results may serve as a basis for clinical assessment of gait and motor control during stair walking in older people. We hypothesize that the 1) the reliability of kinematic and kinetic parameters of stair walking is high under single-task conditions 2) and poor under dual-task conditions.

## 2 Materials and methods

### 2.1 Participants

Fifteen healthy elderly adults (7 women and 8 men, 66.8 ± 3.6 years old, 161 ± 4.3 cm, 61.6 ± 4.2 kg) were recruited from a local community by distributing flyers in Jinan city, China. Some studies have reported that the estimation of the minimum sample size can be calculated based on the reliability of the ICC and the number of measurements of the subject (Donner and Eliasziw, 1987). A previous study found that the test-retest reliability was good to excellent for most models for lower limb kinematics and kinetics (ICC≥0.8) (Mentiplay and Clark, 2018). We set ICC = 0.8, *α* = 0.05, and power = 0.8, and when the number of measurements of the subject is two, according to the model provided by Donner A, the minimum sample size should be fifteen (Donner and Eliasziw, 1987). All the participants signed an informed consent form. This study was approved by the medical ethics committee of Shandong Sport University (No. 2017103). Participants met the following criteria for inclusion: able to ascend and descend stairs independently; able to understand verbal and written information; medically stable with no other diseases that significantly influenced gait performance (Flansbjer et al., 2005); and Body Mass Index of 18.5–25 kg/m^2^. Exclusion criteria included lower extremity contractures or pain (Dobkin et al., 2011); musculoskeletal or cardiovascular pathologies (Hashish et al., 2017); and Mini Mental State Examination score (MMSE) of <24 points (Lopez et al., 2005).

### 2.2 Data collection

**FIGURE 1:**
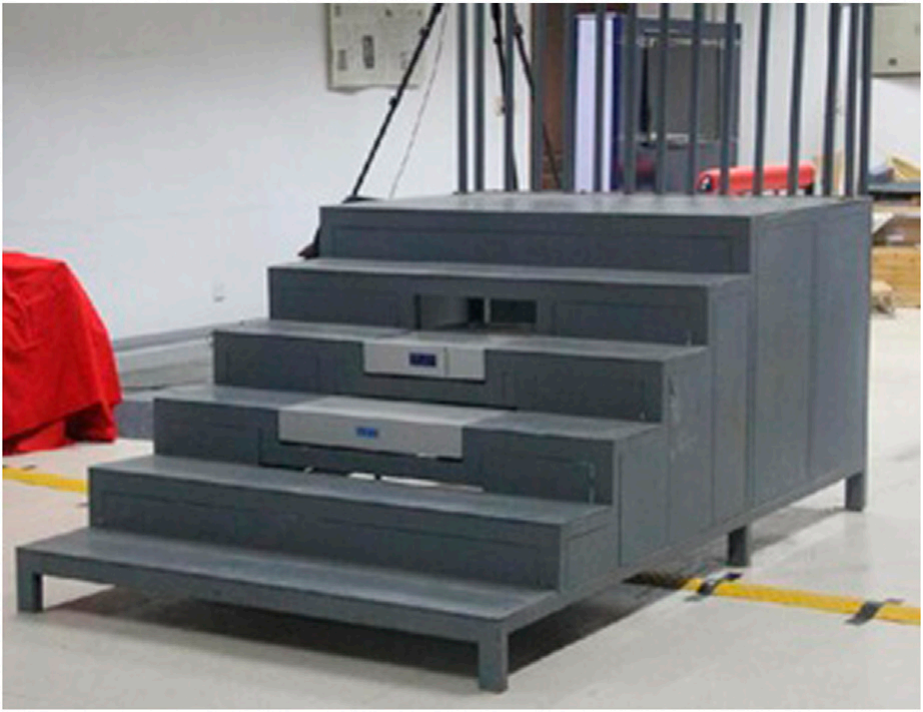
The figure of simulated stairs.

Data were collected in the Biomechanical Laboratory of the Shandong Sport University. Each participant completed the stair walking test in two sessions on two separate days with a one-week interval. The tests were performed by the same experimenter at the same time of day in the same laboratory. A simulated staircase (17 cm riser, 29 cm tread) with six steps was constructed for data collection, which met the national residential standards (Figure 1). Two force platforms (Switzerland, Kistler 9287BA and 9281CA, 90 cm × 60 cm×10 cm) were used to collect kinetic data at 1,000 Hz and embedded in the third and fourth steps of the simulated staircase (Komnik et al., 2018). An infrared motion analysis system with 12 cameras (Vicon, Oxford Metrics Ltd., Oxford, United Kingdom) was used to collect kinematic data at 100 Hz (Komnik et al., 2018; Bates et al., 2021). Kinematic and kinetic data acquisition was synchronized using Vicon Nexus software. Prior to data collection, the participants were asked to wear unified black tight-fitting clothes and shoes and anthropometric data were collected (Law et al., 2021). The dominant leg was determined as the preferred leg for kicking a football. Forty-one reflective markers (14 mm) were placed on each participant’s anatomical landmarks based on the Visual 3D model, including four markers on the head, two on the trunk, 12 on the upper extremities, five on the pelvis, and 18 on the lower extremities. All the participants were instructed to ascend and descend the staircase with the dominant foot for the first step in a step-by-step manner at a comfortable pace under single-task, cognitive task, and manual-task conditions in random order. In the single-task condition, the participants descend and ascend stairs without cognitive or manual task interference. For the cognitive-task condition, the participants descend and ascend the stairs while performing subtraction of serial threes from a random three-digit number. For the manual-task condition, the participants descend and ascend the stairs while carrying a glass of water (0.63 kg) with the dominant hand. No instruction to prioritize the tasks were given to eliminate interference. Five trials were collected for each condition with a 1-min break between consecutive trials.

### 2.3 Data processing

When ascending the stairs, a stride cycle was defined as the first foot contact on the second step and ended at the same foot contact on the fourth step. When descending the stairs, the selected stride cycle started with the foot contact (of the same foot) on the fourth step and ended with the foot contact (of the same foot) on the second step (Protopapadaki et al., 2007). The first double-limb support phase was defined as when the right foot touched down until the left foot took off (Gorelick et al., 2009; Buckley et al., 2018). The single-limb support phase was defined as when the left foot took off until the left foot touched down. The second-double limb support phase was defined as when the left foot touched down until the right foot took off. The swing phase was defined as when the right foot took off until the right foot touched down (Singhal et al., 2014; Quintero et al., 2015). Vicon Nexus data were used to command, denoise the infrared reflective markers, and delete the markers in static and dynamic acquisition data. We completed the data by interpolation and intercepted the action frame number according to the gait cycle definition, renamed the intercepted data, and exported it to the C3d file. The exported static and dynamic c3d files were imported to Visual 3D for in-depth data processing and index extraction. A static model was built using the files and connected with the dynamic data. After selecting the data, the kinematic and kinetic signals were low-pass filtered by Pipeline with a cutoff frequency of 6 Hz (Singhal et al., 2014).

### 2.4 Parameters

#### 2.4.1 Kinematic parameters

Step cadence was expressed as the number of steps in a minute (steps/min). Step speed was obtained by dividing the stride cycle duration into the displacement of the center of mass (m/s) (Buckley et al., 2018). Step length was expressed as the distance between the heel of the right foot during stair walking from the landing of the right foot to the landing again(m). Step width was defined as the medial–lateral distance between the midpoints of each foot(m) (Baudendistel et al., 2020).

#### 2.4.2 Kinetic parameters

The net moment of the hip–knee–ankle joint was obtained based on the inverse dynamics calculation formula. Specific settings were used in V3d software to select the mechanical parameters to be calculated, the lower limb joint, and the reference link to complete the calculation of the net moment of the lower limb joint. In this study, the flexion and extension moments of the joint in the sagittal plane around the *x*-axis were selected for analysis. Joint movement was expressed as the peak flexion and extension moment of the hip, knee, and ankle joint in the sagittal plane (N·m/kg).

### 2.5 Statistical analysis

The kinematic and kinetic data were analyzed by SPSS (version 25.0) and presented as mean and standard deviation. Test—retest relative reliability was assessed using the intraclass correlation coefficient (ICC) test and interpreted as follows: poor (0 < ICC <0.39), fair (0.40 ≤ ICC <0.59), good (0.60 ≤ ICC < 0.74), and excellent (0.75 ≤ ICC < 1.0) (Cicchetti and Sparrow, 1981). Pearson correlation analysis was used to assess the linear correlation of the data from the two tests. The relationship was assessed as: small (0–0.3), moderate (0.3–0.5), significant (0.5–0.8), and highly relevant (0.8–1.0). The Bland–Altman method was used to assess the distribution of the mean and the difference of the two test data sets by using a scatter plot of the differences (Aandstad and Simon, 2013). Statistical significance was set at *p* < 0.05 (Clemons and Harrison, 2008).

## 3 Results

### 3.1 Test-retest reliability of kinematic and kinetic parameters during ascending stairs

Table 1 shows that the interclass correlation coefficient values ranged from 0.909 to 0.963 in step cadence, from 0.866 to 0.942 in step speed, from 0.394 to 0.553 in step length, and from 0.511 to 0.773 in step width under the three task conditions. The relationship values ranged from 0.840 to 0.929 in step cadence, from 0.768 to 0.891 in step speed, from 0.203 to 0.246 in step length, and from 0.344 to 0.630 in step width under the three conditions.

**TABLE 1:** The test-retest reliability of kinematic parameters under three conditions when ascending stairs.

		First session	second session	ICC	95% CI	r	P
Single task	Step cadence (step/min)	97.4 ± 11.5	98.0 ± 9.8	0.936	0.870–0.969	0.894	<0.001
	Step speed (m/s)	0.45 ± 0.05	0.47 ± 0.05	0.866	0.726–0.935	0.768	<0.001
	Step length(m)	0.58 ± 0.02	0.59 ± 0.02	0.394	−0.242–0.704	0.246	0.175
	Step width(m)	0.07 ± 0.03	0.08 ± 0.04	0.511	−0.002–0.761	0.344	0.054
Cognitive task	Step cadence	83.9 ± 11.6	83.5 ± 13.2	0.909	0.813–0.955	0.840	<0.001
	Step speed (m/s)	0.40 ± 0.06	0.40 ± 0.07	0.907	0.809–0.954	0.840	<0.001
	Step length(m)	0.58 ± 0.01	0.58 ± 0.01	0.553	−2.181–0.242	0.219	0.229
	Step width(m)	0.09 ± 0.04	0.07 ± 0.04	0.773	0.535–0.889	0.630	<0.001
Manual task	Step cadence (step/min)	85.2 ± 12.4	85.99 ± 12.35	0.963	0.924–0.982	0.929	<0.001
	Step speed (m/s)	0.40 ± 0.05	0.41 ± 0.05	0.942	0.881–0.972	0.891	<0.001
	Step length(m)	0.57 ± 0.01	0.58 ± 0.02	0.500	−2.072–0.268	0.203	0.266
	Step width(m)	0.08 ± 0.05	0.07 ± 0.04	0.741	0.470–0.874	0.594	<0.001

Table 2 shows that the interclass correlation coefficient values ranged from 0.858 to 0.922 in min hip moment, from 0.881 to 0.954 in maximum knee moment, and from 0.857 to 0.979 in minimum ankle moment under the three task conditions. The relationship values ranged from 0.751 to 0.856 in minimum hip moment, from 0.787 to 0.914 in maximum knee moment, and from 0.764 to 0.960 in minimum ankle moment under the three conditions.

**TABLE 2:** The test-retest reliability of kinetic parameters under three conditions when ascending stairs. (N.m/kg).

		First session	second session	ICC	95% CI	r	P
Single task	Min hip moment	0.91 ± 0.17	0.90 ± 0.17	0.872	0.738–0.938	0.773	<0.001
	Max knee moment	0.72 ± 0.18	0.72 ± 0.19	0.881	0.755–0.942	0.787	<0.001
	Min ankle moment	1.25 ± 0.29	1.25 ± 0.28	0.979	0.957–0.990	0.960	<0.001
Cognitive task	Min hip moment	0.91 ± 0.17	0.91 ± 0.17	0.858	0.709–0.931	0.751	<0.001
	Max knee moment	0.70 ± 0.18	0.70 ± 0.16	0.954	0.906–0.978	0.914	<0.001
	Min ankle moment	1.21 ± 0.15	1.21 ± 0.15	0.889	0.773–0.946	0.800	<0.001
Manual task	Min hip moment	0.81 ± 0.15	0.78 ± 0.15	0.922	0.840–0.962	0.856	<0.001
	Max knee moment	0.82 ± 0.19	0.83 ± 0.18	0.917	0.830–0.959	0.848	<0.001
	Min ankle moment	1.21 ± 0.18	1.20 ± 0.15	0.857	0.706–0.930	0.764	<0.001

**FIGURE 2:**
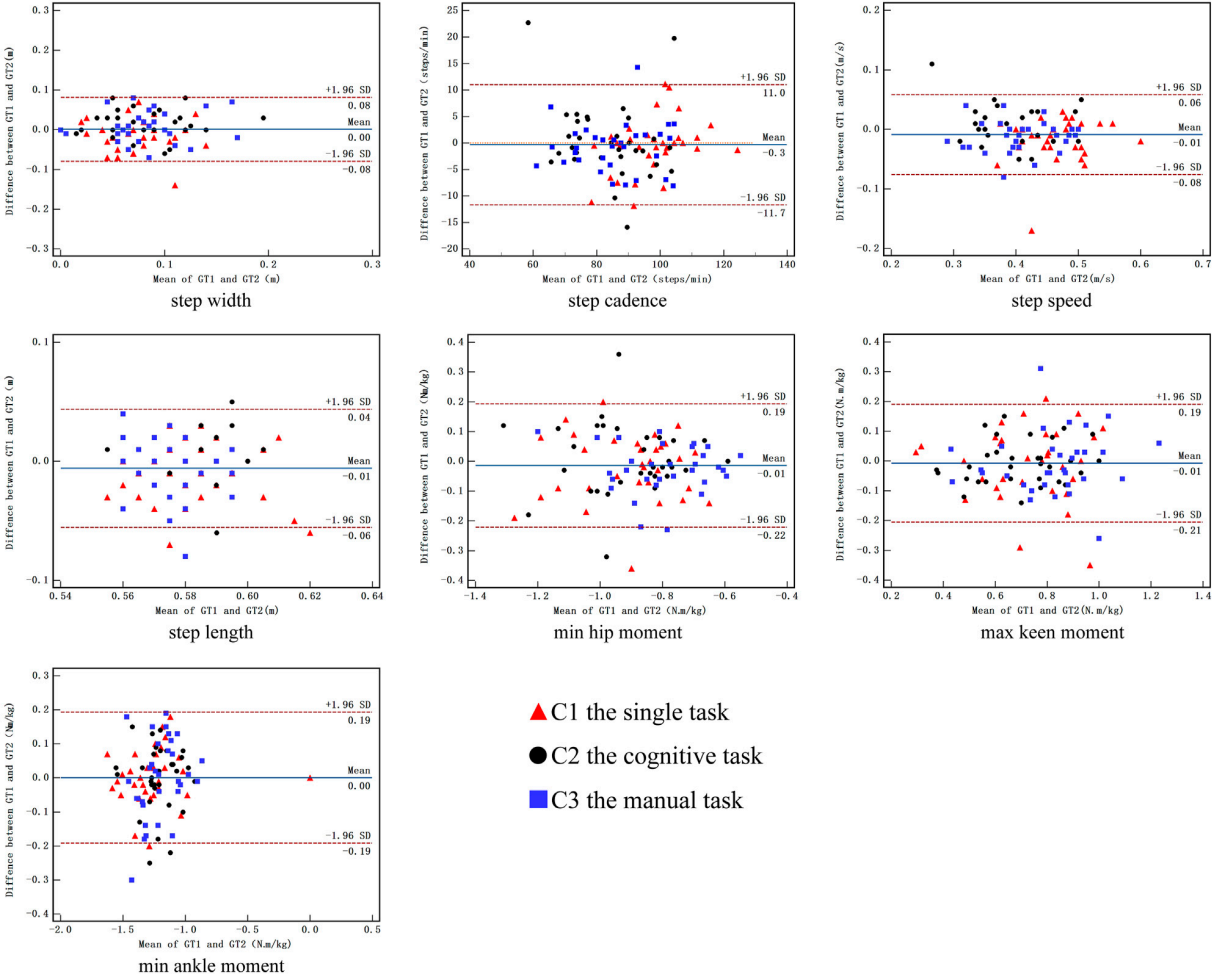
Bland-Altman plot of the difference between session 1 and session 2 against mean value during ascending stair for kinematic and kinetic parameters. C1, the single task; C2 the cognitive task; C3 the manual task.

Figure 2 shows the Bland—Altman plots of the kinematic and kinetic parameters when ascending stairs. The systematic basis around the zero line for kinematic and kinetic parameters was revealed. All the zero values fell in the 95% confidence interval. The numbers of dots inside the range of 95% CI interpreted the reliability. A higher number of dots indicated better reliability. In this study, the numbers of dots outside the range of 95% CI were one dot (3.1%) in step width to two dots (6.3%) in step cadence in the single task, three dots (9.4%) in step cadence in the cognitive task, and one dot (3.1%) in step cadence in the manual task. The numbers of dots outside the range of 95% CI were one dot (3.1%) in step speed to two dots (6.3%) in step length in the single task, from one dot (3.1%) in step speed to two dots (6.3%) in step length in the cognitive task, and one dot (3.1%) in step speed and step length in the manual task. The numbers of dots outside the range of 95% CI were two dots (6.3%) in minimum hip moment to three dots (9.4%) in maximum knee moment in the single task, two dots (6.3%) in minimum hip moment in the cognitive task, and one dot (3.1%) in minimum hip moment to two dots (6.3%) in maximum knee moment in the manual task. The numbers of dots outside the range of 95% CI were one dot (3.1%) in minimum ankle moment in the single task, two dots (6.3%) in minimum ankle moment in the cognitive task, and one dot (3.1%) in minimum ankle moment in the manual task.

### 3.2 Test—retest reliability of kinematic and kinetic parameters during descending stairs

Table 3 shows that the interclass correlation coefficient values ranged from 0.925 to 0.956 in step cadence, from 0.779 to 0.932 in step speed, from 0.305 to 0.745 in step length, and from 0.661 to 0.795 in step width under the three task conditions. The relationship values ranged from 0.866 to 0.916 in step cadence, from 0.639 to 0.873 in step speed, from 0.185 to 0.597 in step length, and from 0.500 to 0.660 in step width under the three conditions.

**TABLE 3:** The test-retest reliability of kinematic parameters under three conditions when descending stairs.

		Firs session	Secon session	ICC	95% CI	r	P
Single task	Step cadence (step/min)	109.1 ± 16.1	109.2 ± 16.6	0.956	0.909–0.978	0.916	<0.001
	Step speed (m/s)	0.56 ± 0.08	0.55 ± 0.09	0.878	0.750–0.940	0.785	<0.001
	Step length(m)	0.63 ± 0.02	0.63 ± 0.02	0.745	0.478–0.876	0.597	<0.001
	Step width(m)	0.07 ± 0.04	0.07 ± 0.04	0.795	0.580–0.900	0.660	<0.001
Cognitive task	Step cadence	90.4 ± 19.4	91.9 ± 19.8	0.944	0.886–0.973	0.895	<0.001
	Step speed (m/s)	0.48 ± 0.10	0.47 ± 0.10	0.932	0.861–0.967	0.873	<0.001
	Step length(m)	0.62 ± 0.02	0.62 ± 0.02	0.710	0.597–0.885	0.396	0.025
	Step width(m)	0.08 ± 0.03	0.08 ± 0.04	0.685	0.354–0.846	0.528	0.002
Manual task	Step cadence	91.4 ± 13.7	93.6 ± 15.4	0.925	0.846–0.963	0.866	<0.001
	Step speed (m/s)	0.47 ± 0.08	0.47 ± 0.08	0.779	0.548–0.892	0.639	<0.001
	Step length(m)	0.62 ± 0.02	0.62 ± 0.02	0.305	−4.25–0.661	0.185	0.310
	Step width(m)	0.06 ± 0.04	0.06 ± 0.03	0.661	0.306–0.835	0.500	0.004

Table 4 shows that the interclass correlation coefficient values ranged from 0.133 to 0.963 in minimum hip moment, from 0.893 to 0.935 in maximum knee moment, and from 0.057 to 0.850 in minimum ankle moment under the three task conditions. The relationship values ranged from 0.075 to 0.930 in minimum hip moment, from 0.816 to 0.887 in maximum knee moment, and from 0.030 to 0.752 in minimum ankle moment under the three conditions.

**TABLE 4:** The test-retest reliability of kinetic parameters under three conditions when descending stairs. (N m/kg).

		First session	Secon session	ICC	95% CI	r	P
Single task	Min hip moment	0.54 ± 0.28	0.58 ± 0.30	0.963	0.924–0.982	0.930	<0.001
	Max knee moment	1.13 ± 0.25	1.08 ± 0.29	0.935	0.867–0.968	0.887	<0.001
	Min ankle moment	1.03 ± 0.19	1.07 ± 0.25	0.811	0.612–0.908	0.706	<0.001
Cognitive task	Min hip moment	0.55 ± 0.32	0.55 ± 0.28	0.958	0.913–0.979	0.928	<0.001
	Max knee moment	1.10 ± 0.24	1.14 ± 0.28	0.929	0.855–0.965	0.878	<0.001
	Min ankle moment	1.01 ± 0.25	1.04 ± 0.21	0.850	0.693–0.927	0.752	<0.001
Manual task	Min hip moment	0.50 ± 0.28	0.47 ± 0.20	0.133	−0.776–0.577	0.075	0.683
	Max knee moment	1.09 ± 0.23	1.11 ± 0.27	0.893	0.780–0.948	0.816	<0.001
	Min ankle moment	1.01 ± 0.19	0.98 ± 0.23	0.057	−0.933–0.539	0.030	0.871

**FIGURE 3:**
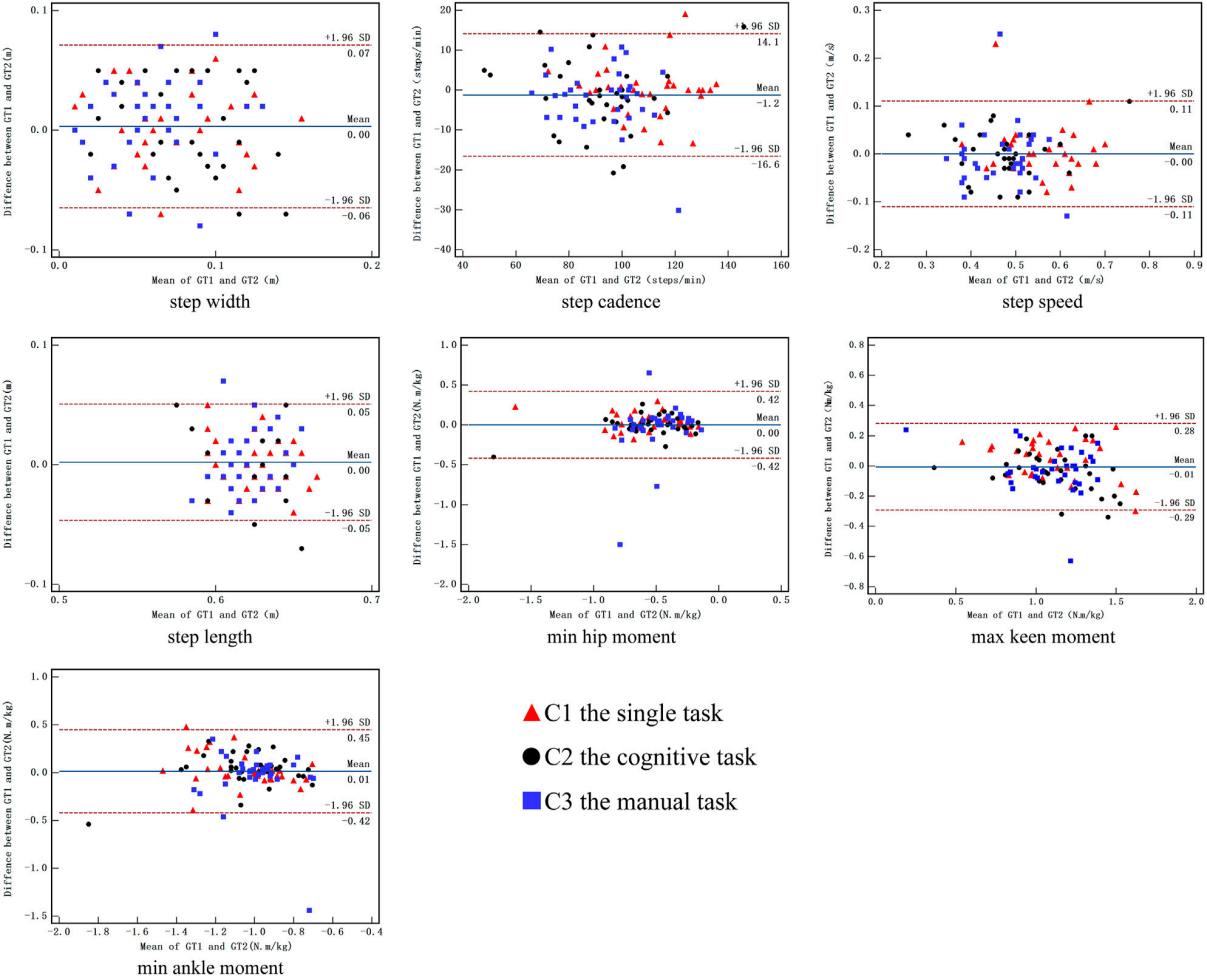
Bland-Altman plot of the difference between session 1 and session 2 against mean value during descending stair for kinematic and kinetic parameters. C1, the single task; C2 the cognitive task; C3 the manual task.

Figure 3 shows the Bland—Altman plots of the kinematic and kinetic parameters during descending stairs. The numbers of dots outside the range of 95% CI were one dot (3.1%) in step width and step cadence in the single task, two dots (6.3%) in step width to four dots (12.5%) in step cadence in the cognitive task, and three dots (9.4%) in step width to one dot (3.1%) in step cadence in the manual task. The numbers of dots outside the range of 95% CI were one dot (3.1%) in step speed in the single task, two dots (6.3%) in step length in the cognitive task, and two dots (6.3%) in step speed to one dot (3.1%) in step length in the manual task. The numbers of dots outside the range of 95% CI were one dot (3.1%) in maximum knee moment in the single task, two dots (6.3%) in maximum knee moment in the cognitive task, and one dot (3.1%) in maximum knee moment to three dots (9.4%) in minimum hip moment in the manual task. The numbers of dots outside the range of 95% CI were one dot (3.1%) in minimum ankle moment in the single task and cognitive task and two dots (6.3%) in minimum ankle moment in the manual task.

## 4 Discussion

This study revealed the test—retest reliability of kinematic and kinetic parameters in the elderly during stair walking under single task, cognitive task, and manual task conditions (Flansbjer et al., 2005; Gorelick et al., 2009). Most of the test–retest reliability values for these parameters were good to excellent.

ICC is a popular statistical method used to assess test–retest reliability (Chan and Pin, 2019) and is based on the calculation of the F-value from repeated-measures ANOVA. In this study, most of the test–retest reliability values of the kinematic and kinetic parameters were high in the single task, which supported the first hypothesis. The ICC value (0.511–0.979) demonstrated fair to excellent test–retest reliability in the single task, except for step length (Flansbjer et al., 2005). The finding was consistent with the study of Santos et al. (Santos et al., 2022), who reported that the agreement between manual stopwatches and photoelectric cells was excellent (ICCs between 0.92 and 0.97) when the distance was 5 m. Bland—Altman plot analysis is an important method used to detect systematic errors (Bland and Altman, 1999). The axis of ordinate was determined based on the differences between two measurements, and the axis of abscissa was determined based on the mean of two measurements. In some clinical studies, Bland—Altman analysis was used to evaluate the agreement between two testing sessions (Soulard et al., 2021). Reliability was assessed using the 95% limits of agreement and mean difference. As shown in Figures 2, 3, during single task, the zero values were included in the 95% CI, few dots were outside the range of 95% CI, and the mean difference was close to zero, indicating fair reproducibility (Dolatabadi et al., 2016). The good test–retest reliability of the kinematic and kinetic parameters during single-task stair walking might be attributed to several factors. First, the same testing control could help reduce measurement errors, including the same time interval between consecutive tests, the same testing commands, the same gait and postural control during stair walking, and the laboratory environment (Flansbjer et al., 2005). Second, three pre-test practices were provided to each participant before data collection to help them understand the procedure and minimize errors in learning (Sun et al., 2015). Finally, the test and retest sessions of reliability were scheduled a week apart, which could prevent learning and fatigue from affecting the reproducibility of the measurements.

In dual-task stair walking, the ICC and r values of the kinematic and kinetic parameters demonstrated fair to excellent test–retest reliability. The Bland–Altman plot also showed that the zero values were included in the 95% CI, the mean difference was close to zero, and about 14 dots (87.5%) were inside the range of 95% CI during cognitive and manual stair walking. Our results showed that the test–retest reliability of the kinematic and kinetic parameters during dual-task stair walking was fair to excellent, which did not support the second hypothesis. However, previous studies were in line with our results. A recent study by Cho et al. showed that some level walking gait parameters (e.g., speed, stride length, cadence, etc.) while talking had good reliability (ICC = 0.69–0.90) under dual-task conditions (Cho et al., 2015). Tsang et al., (2019) also found that the test–retest reliability of the dual-task walking distance tests were excellent (ICC = 0.891–0.984). When confronted by two attention-demanding activities, humans prioritize one task over the other based on counterbalancing capabilities and cognitive availability (Yogev-Seligmann et al., 2012). The elderly might prefer to perform posture control of gait stability rather than deal with the ‘‘secondary’’ cognitive task during walking to minimize the loss of balance and maximize the avoidance of hazards (Shumway-Cook et al., 1997). When facing more complexed situations, such as stair walking, individuals tend to allocate attention resources for posture control to ensure safety (Wang et al., 2021). Therefore, the test–retest reliability of posture control during stair walking is high.

However, the ICC value ranged from 0.394 to 0.553, and the r value ranged from 0.203 to 0.246 of step length when ascending stairs under single- and dual-task conditions, indicating the poor test–retest reliability of step length. The ICC values were 0.133 in minimum hip moment and 0.057 in minimum ankle moment; the r values were 0.075 in minimum hip moment and 0.030 in minimum ankle moment in the manual task when descending stairs, which showed the poor test–retest reliability of both parameters. Our finding was inconsistent with the results of this study (Ryan et al., 2006); at all speeds, inter device reliability was excellent for the activPAL physical activity monitor ((ICC (2,1)≥0.99)) for both step number and cadence. The discrepancy might be related to the differences in gait complexion between stair and level walking. More challenging and complex tasks might require more attentional resources (Ruffieux et al., 2015). Stair walking, as the most hazardous aspect of gait, might be more challenging and complex and requires greater attentional resources (Mian et al., 2007). According to capacity–sharing theory, cognitive resources are limited in capacity; when two tasks sharing common neural circuitry are performed at the same time, both of them are processed, but the processing ability between the tasks will decrease (Patel et al., 2014). When performing stair walking and dual tasks simultaneously (Wang et al., 2021), the poor test–retest reliability may have occurred due to the competition of limited attention resources, resulting in the decline of task performance in dual-task situation during stair walking (Ettwig and Bronkhorst, 2015). This study might provide reference for the selection of kinematic and kinetic parameters during dual-task stair walking.

This study has three limitations. First, the sample size was relatively small, and the test–retest reliability of stair walking should be used cautiously for clinical assessment. Second, all the participants were healthy elderly adults, and further studies should focus on the reliability of stair walking in frail elderly adults with a high fall risk. Finally, we only recruited people with a BMI of 18.5–25 Kg/m^2^, so the result should be cautiously used for the elderly population.

## 5 Conclusion

The results obtained from this study show the good test-retest reliability of step cadence, step speed, and step width during single- and dual-task stair walking in the elderly, and the poor reliability of step length when ascending stairs. All the kinetic parameters, including min hip moment, max knee moment, and min ankle moment, had good test-retest reliability during single- and dual-task stair walking, but min hip moment and min ankle moment had poor reliability during manual-task descending stairs. These results may help researchers in the assessment of biomechanics of dual-task stair walking in the elderly and to interpret the effect of interventions in this population.

## Data Availability

The original contributions presented in the study are included in the article/supplementary material, further inquiries can be directed to the corresponding author.
